# Understanding the motivations of health-care providers in performing female genital mutilation: an integrative review of the literature

**DOI:** 10.1186/s12978-017-0306-5

**Published:** 2017-03-23

**Authors:** Marie-Hélène Doucet, Christina Pallitto, Danielle Groleau

**Affiliations:** 1 Division of Social and Transcultural Psychiatry, McGill University, 1033, Des Pins West, Montreal, QC H3A 1A1 Canada; 2 Department of Reproductive Health and Research, World Health Organization, Avenue Appia 20, Geneva, 1201 Switzerland; 3 Jewish General Hospital, Lady Davis Institute, 4333 Côte St-Catherine Road, Montreal, QC H3T 1E4 Canada

**Keywords:** Female genital mutilations (FGM), Re-infibulation, Health-care providers, Medicalization, Motivating factors, Mutilations génitales féminines (MGF), Réinfibulation, Personnel de santé, Médicalisation, Motivations

## Abstract

**Background:**

Female genital mutilation (FGM) is a traditional harmful practice that can cause severe physical and psychological damages to girls and women. Increasingly, trained health-care providers carry out the practice at the request of families. It is important to understand the motivations of providers in order to reduce the medicalization of FGM. This integrative review identifies, appraises and summarizes qualitative and quantitative literature exploring the factors that are associated with the medicalization of FGM and/or re-infibulation.

**Methods:**

Literature searches were conducted in PubMed, CINAHL and grey literature databases. Hand searches of identified studies were also examined. The “CASP Qualitative Research Checklist” and the “STROBE Statement” were used to assess the methodological quality of the qualitative and quantitative studies respectively. A total of 354 articles were reviewed for inclusion.

**Results:**

Fourteen (14) studies, conducted in countries where FGM is largely practiced as well as in countries hosting migrants from these regions, were included. The main findings about the motivations of health-care providers to practice FGM were: (1) the belief that performing FGM would be less harmful for girls or women than the procedure being performed by a traditional practitioner (the so-called “harm reduction” perspective); (2) the belief that the practice was justified for cultural reasons; (3) the financial gains of performing the procedure; (4) responding to requests of the community or feeling pressured by the community to perform FGM. The main reasons given by health-care providers for *not* performing FGM were that they (1) are concerned about the risks that FGM can cause for girls’ and women’s health; (2) are preoccupied by the legal sanctions that might result from performing FGM; and (3) consider FGM to be a “bad practice”.

**Conclusion:**

The findings of this review can inform public health program planners, policy makers and researchers to adapt or create strategies to end medicalization of FGM in countries with high prevalence of this practice, as well as in countries hosting immigrants from these regions. Given the methodological limitations in the included studies, it is clear that more robust in-depth qualitative studies are needed, in order to better tackle the complexity of this phenomenon and contribute to eradicating FGM throughout the world.

**Electronic supplementary material:**

The online version of this article (doi:10.1186/s12978-017-0306-5) contains supplementary material, which is available to authorized users.

## Résumé

**Contexte:**

Les mutilations génitales féminines (MGF) peuvent gravement affecter la santé physique et psychologique des filles et des femmes. De plus en plus de professionnels formés pour donner des soins de santé pratiquent les MGF à la demande des familles. Il importe de connaître les motivations de ceux-ci afin de réduire la médicalisation des MGF. Cette revue intégrative identifie, évalue et résume la littérature scientifique qualitative et quantitative explorant les facteurs associés à la médicalisation des MGF ou de la ré-infibulation.

**Méthodes:**

Les recherches ont été menées dans PubMed, CINAHL et des bases de données de la littérature grises. Les références des études identifiées ont aussi été examinées. Les listes de vérification du CASP et de l’énoncé STROBE ont été utilisées pour évaluer la qualité méthodologique des études qualitatives et quantitatives. Au total, 354 articles ont été révisés pour inclusion.

**Résultats:**

Quatorze études, menées dans des pays où les MGF sont pratiquées et dans des pays accueillant des migrants, ont été incluses. Les principales motivations nommées par le personnel de santé pour pratiquer les MGF étaient: (1) croire que cela réduira les risques pour la santé des filles, par opposition à l’exécution par une exciseuse traditionnelle (“réduction des méfaits”); (2) pratiquer pour des raisons culturelles; (3) bénéficier de gains financiers; (4) répondre aux demandes ou ressentir une pression provenant de la communauté pour effectuer les MGF. Les principales raisons pour lesquelles certains professionnels de la santé ne pratiquent *pas* les MGF étaient qu’ils (1) sont préoccupés par les risques encourus pour la santé des filles et des femmes; (2) craignent des sanctions légales; et (3) considèrent les MGF comme étant une “mauvaise pratique”.

**Conclusion:**

Les résultats de cette recension permettent d’informer les planificateurs de santé publique, les décideurs et les chercheurs, afin d’adapter ou de créer des stratégies permettant de mettre fin à la médicalisation des MGF dans les pays à forte prévalence, ainsi que dans les pays qui accueillent les immigrants de ces régions. Cependant, étant donné les limites méthodologiques importantes des études incluses, des études qualitatives plus robustes sont requises, afin de mieux lutter contre ce phénomène complexe et contribuer à éradiquer les MGF dans le monde.

## Plain English summary

Female genital mutilation (FGM) is a traditional harmful practice involving the cutting or removal of flesh from girls’ genitals, and sometimes stitching the vagina closed. In addition to being a violation of human rights, the practice increases risk of severe harm to girls and women, such as sexual problems, complications during childbirth, psychological problems and even death. While mainly performed by traditional practitioners, there is an increasing trend of trained health-care providers performing FGM. This review seeks to identify the reasons why health-care providers perform FGM, or not. The main reasons identified are (1) the belief that it will reduce risks for girls or women, as compared to when it is done by a traditional practitioner; (2) for cultural reasons; (3) for financial gain; (4) to respond to the requests of families and community members. The main reasons why health-care providers do *not* perform FGM are that (1) they consider FGM as being a bad practice; (2) they are concerned about the risks that FGM can cause for girls’ and women’s health; and (3) they are preoccupied by the legal sanctions that might result from performing FGM. These findings can contribute to the development of strategies to end the practice of FGM by health-care providers. In addition, there is a need for more research on best approaches to reduce the medicalization of FGM.

## Background

### Female genital mutilation

Female genital mutilation (FGM) is an ancient tradition, practiced in at least 30 countries of Africa, the Middle-East and Asia [1]. It is estimated that there are currently 200 million women and girls living with FGM [1], and that more than 3 million girls are at risk of being cut every year [2]. FGM involves the partial or total removal of the external female genitalia, or other injury to the female genital organs for non-medical reasons [3]. Different forms of FGM include clitoridectomy (partial or total removal of the clitoris, referred to as Type 1), excision (partial or total removal of the clitoris and the labia minora, with or without excision of the labia majora, Type 2), and other forms such as pricking, piercing, incising, scraping and cauterizing the genitals (Type 4). The most severe form of FGM (Type 3) also known as infibulation involves the removal of the clitoris and labia minora, and stitching closed of the labia majora [4]. Women who have undergone Type 3 may have a procedure called de-infibulation, which involves opening the infibulation scar in order to facilitate childbirth or to prevent complications from the infibulation. Some women or their families request re-infibulation after childbirth to restore the genitalia to the state that they were in as a result of infibulation [3]. Immediate risks of FGM include intense pain, haemorrhage (excessive bleeding), shock, difficult urination and infection [5]. In the long term, FGM can also give rise to reproductive health problems, such as dyspareunia (difficulty having sexual intercourse), complications during childbirth and even neonatal death, particularly among women who have been infibulated [6, 7]. Moreover, psychological health problems such as anxiety, depression and post-traumatic stress disorder are associated with this practice [8, 9]. Furthermore, some girls and women do not survive the complications of FGM such as haemorrhage, infections (e.g., tetanus) and obstructed labour [2, 10, 11]. Lastly, FGM yields no health benefit [4]. Hence, these practices represent an important public health problem, and a violation of the fundamental rights to security, health and life [4, 12, 13].

The prevalence of FGM varies from one region to another, and is for example almost universal in Somalia (98%) and Guinea (97%), very high in Mali (89%), Egypt and Sudan (87%), relatively low in Senegal (25%) and almost non-existent in Cameroon (1%) [1]. FGM is usually practiced on girls younger than 15 years old [2]. The reasons given to justify this custom are diverse, and mainly reflect cultural and social dimensions [4, 14], including cultural ideals of beauty and “cleanliness”, and are a pivotal part of the rite of passage to adulthood [2, 14, 15]. As it is rooted in gender inequality, FGM is intended to control women’s sexuality and safeguard the honour of the family [2, 5] by ensuring virginity among young girls and marital fidelity among married women [2, 16]. In addition, FGM would confer girls the status of eligibility for marriage [2]. In communities where FGM is nearly universal, mothers may not question the practice for their daughters [2, 15]. FGM tends to be practiced more in rural areas compared to urban areas [2], with the ethnicity being the most important factor predicting the prevalence and type of FGM performed [2]. Despite multiple international resolutions and declarations on ending FGM [17, 18], and diverse strategies throughout the world to eradicate it, the practice of FGM persists [1]. This stems from the fact that the cultural beliefs associated with FGM are central for practicing communities, which do not consider FGM as being a form of violence or a “mutilation” [19, 20], and consequently not as a violation of human rights. On the contrary, they believe FGM is necessary: indeed, families have their daughters cut with the intention of providing them with a viable future [19]. Moreover, FGM is a complex sociocultural phenomenon, and families are generally under great pressure to have their daughters conform to the social norm [2, 21]. Therefore, any strategy addressing FGM should protect human rights in a culturally appropriate manner, in order to be respectful to human beings as well as to prevent policies, programmes or procedures that could inadvertently cause harm.

### The medicalization of female genital mutilation: a “new” phenomenon

According to the WHO definition, when FGM is performed by any category of health-care provider, it is referred to as “medicalization of FGM”, which includes the practice of any type of FGM, as well as re-infibulation, performed regardless of the setting (i.e., either public or private, in clinic, at home or elsewhere) [3].

*Who performs FGM?* FGM is mainly performed by traditional practitioners (traditional circumcisers or traditional birth attendants). However, in recent years, there has been a dramatic increase in the proportion of FGM carried out by health-care providers (defined in the present review as trained medical doctors, nurses and midwives) in many settings [2]. Indeed, families are increasingly requesting that health-care providers perform FGM, based on the belief that it would prevent health consequences for girls [2, 22]. This phenomenon is thought to be at least partially a result of the awareness campaigns about the risks of FGM for the health of girls [3, 23]. In fact, most traditional practitioners use an unsterilized blade or a razor to perform the cut [2]. Moreover, they generally do not have adequate knowledge about the anatomy and physiology of the human body and the principles of infection prevention, nor the training to treat the consequences of FGM [24]. However, even when performed with sterile instruments by trained providers, FGM is not without risk, and the removal of healthy body parts can result in adverse consequences in the short- and long-term [3, 25]. Moreover, health-care providers are generally respected members of the community, and when they practice FGM, this can give the impression that the procedure is acceptable and safe, which can further promote the practice. Since FGM is performed for sociocultural reasons rather than for medical reasons, the practice goes against the Hippocratic Oath of “Do no harm”, and it violates girls’ and women’s right to physical integrity, health and life. Therefore, the World Health Organization (WHO), in its *Global strategy to stop health-care providers from performing female genital mutilation* [3], condemns the practice of FGM by health-care providers, or by anyone else.

*Why do health-care providers perform FGM?* In order to be able to address the issue of medicalization, it is essential to understand the perspective of health-care providers. Some studies have asked providers whether they were requested to perform FGM or re-infibulation [26, 27], but few studies explored the reasons why or why not they agreed to do so. To our knowledge, only one review [28] has attempted to assess the reasons for which health-care providers practice FGM. However, this review only focused on medical doctors. In addition, it found only one study that addressed this objective. No review was found on the reasons why or why not nurses and midwives perform FGM, including re-infibulation. Therefore, this review fills a gap by identifying, appraising and summarizing qualitative and quantitative evidence on the motivations of different types of health-care providers (nurses, midwives and medical doctors) and of future health-care providers (students of these disciplines) to perform FGM and/or re-infibulation. This knowledge will mainly inform public health program planners, policy makers and researchers for adapting or creating strategies to end medicalization of FGM in high prevalence countries, as well as in countries with migrant populations from these countries.

## Methods

### Search strategy

To identify any qualitative or quantitative research on the medicalization of FGM, an integrative review method was used. Indeed, the “integrative review method is an approach that allows for the inclusion of diverse methodologies (i.e. experimental and non-experimental research)” [29]. A systematic search strategy was developed for PubMed and CINAHL databases for peer-reviewed articles, using controlled vocabulary and free keywords, combining 2 concepts: (a) female genital mutilation; and (b) health-care providers, including medicalization (Additional file 1). Searches were conducted during March and April 2016 and updated in August 2016. No language restrictions were imposed, but dates were limited to 2001-2016. Additional searches were also performed in Google Scholar, WHO Library & Information Networks for Knowledge Database (WHOLIS), WHO Global Health Library and Open Grey to search for remaining peer-reviewed studies as well as for grey literature, such as research reports produced by non-governmental organizations. EThOS was used to search for PhD theses. Finally, a manual search of the reference list of all included studies, as well as reports [2, 3] and reviews on knowledge, experiences and attitudes of health-care providers about FGM [28, 30] was also conducted.

### Selection of studies

The inclusion criteria used were the following: (1) the study described was a primary study; (2) only recent years (2001–2016) were included, since practices around medicalization have changed in the past 10–15 years; (3) the study appeared in a peer-reviewed journal, in the grey literature from recognized institutions and/or governments, or was a PhD thesis; (4) the population researched included health-care providers of any type (physicians, nurses, or midwives), or students of these professions; (5) the study related to the topic of medicalization, including on the motivations of practicing FGM. There were no restrictions on (a) the methodology: both quantitative or qualitative studies were included; (b) the setting: all were considered (i.e., public practice, private practice, including at the home of the girls or the home of the health-care provider); (c) the countries: studies assessing the practices of health-care providers practicing in regions with high prevalence of FGM or in countries hosting immigrants from high prevalence regions were all examined. Studies were excluded if health-care providers were not the population under study, if there was no mention whether health-care providers performed FGM and/or re-infibulation, or if the reasons for which providers perform FGM and/or re-infibulation (or not) were not reported.

### Quality assessment

An assessment of the methodological quality and limitations of the included studies was undertaken. An enriched version of the “Critical Appraisal Skills Programme (CASP) Qualitative Research Checklist” [31] was used for the qualitative studies. The criteria include the following ten domains, all of which were included in this assessment: the aims of the research, the methodology, the research design, the recruitment strategy, the data collection, the reflexivity of researchers, the ethical considerations, the rigor of data analysis, the findings, and the value of the research. One criterion was added to the list, which pertains to the mention of possible bias or limits of the study, for a total of 34 items (Additional file 2). The checklist of essential items of the “STROBE (Strengthening the Reporting of Observational studies in Epidemiology) Statement” [32] was used to assess the quality of the quantitative studies. This checklist was in fact not designed to appraise quality, but rather to guide researchers for the reporting of observational studies [33]. However, in the absence of a tool designed to judge the methodological quality of surveys, the STROBE Statement was used as a proxy. Moreover, this checklist was modified, since some criteria do not pertain to survey study designs. The 26 included items related to the title, the abstract, the introduction, the methods, the results and the discussion sections of articles. Likewise, a criterion was added to the list pertaining to ethical considerations and more precisely for appraising whether the study was examined and approved by a research ethics committee. The modified version of the checklist comprised a total of 26 sub-items (Additional file 3). Thereby, each study was given a score, which was the number of criteria addressed as a percentage of the total number of applicable items. Any criterion that was met has received a score of 1, an item partially met was marked as 0.5, and a completely absent criterion received the grade of 0. A score of 75% or above was considered as “high” quality, a score of 50–74% reflected “moderate” quality, a score of 25–49% was judged as “low” quality, and a score below 25% was counted as “very low” quality. Because of the paucity of studies found, no study was excluded because of the score; however, the quality scores indicate the level of confidence we can attribute to the findings of this review.

### Data extraction & synthesis

Each study was systematically examined for all relevant information, which was compiled in a matrix. The extracted data included the following domains: year of the publication; country where the study took place; aim of the study; type of study/design; methods used for data collection; type of health-care providers under study; sample size; form of FGM (i.e., Type 1, 2, 3 and/or 4 FGM, and/or re-infibulation) (Table 1). Furthermore, for the qualitative studies, a thematic analysis was conducted, based on the verbatim, results and interpretations reported in the articles. All relevant text units were coded, extracted, and classified in a matrix into two broad categories: “reasons to perform FGM” and “reasons *not* to perform FGM”. The extracted text units were further categorized in an inductive and iterative manner into the themes that emerged from the data. For the quantitative studies, the motivating factors were directly exported into the matrix. At the end of this process, a verification of the extracted themes was undertaken with the primary data, in order to ensure accuracy of the review findings.

**Table 1:** Summary of the articles reviewed (*n* = 14)

Authors (Year)	Country	Aim of the study	Type of study/design	Methods	Sample	n	Form of FGM^a^
Ali [46] (2012)	Sudan	To assess knowledge and attitudes of the midwives towards FGM in Eastern Sudan	Descriptive study	Face to face interview with open questionnaire	Midwives *Note: “midwives used […] were traditional birth attendants”*	157	FGM
Berggren & al. [35] (2004)	Sudan	To explore motives, perceptions and experiences of re-infibulation after birth and to elucidate its context and determinants	Qualitative study (focussed ethnography)	Explorative open-ended interviews	Midwives	17	Re-infibulation
Christof-fersen-Deb [48] (2005)	Kenya	To examine medicalized circumcision from the perspective of Gusii community members and health care workers in western Kenya	Qualitative study	Structured interviews	Medical doctors	4	FGM
					Nurses	7	
Hess & al. [45] (2010)	Unites-States of America	To assess certified nurse-midwives’ knowledge of FGM and to explore their experiences caring for African immigrant women with a history of genital cutting	Descriptive study	Closed and open-ended questionnaire	Nurse-midwives	243	Mainly re-infibulation
Ibrahim & al. [42] (2013)	Nigeria	To determine the knowledge, attitude and practice of FGM among doctors and nurses/midwives practising in public secondary and tertiary hospitals	Cross-sectional descriptive study	Self-administered structured questionnaires	Medical doctors	66	FGM
					Nurses	52	
Kaplan & al. [41] (2013)	The Gambia	To examine the knowledge, attitudes, and practices regarding FGM among health-care providers working in rural settings in The Gambia	Cross-sectional descriptive study	Open and close-ended questions administered face to face	Nurses and Midwives	468	FGM
Leye & al. [44] (2008)	Belgium	To assess the knowledge, attitudes and practices with regard to FGM among gynaecologists in Flanders, Belgium	Descriptive study	Questionnaire	Medical doctors (gynaeco-logists)	333	FGM & Re-infibulation
Mostafa & al. [38] (2006)	Egypt	To explore the knowledge, beliefs and attitudes of medical students; to explore the students’ opinions about the medicalization of FGM	Cross-sectional descriptive study	Structured questionnaires	Medical students	298	FGM
Njue & Askew [47] (2005)	Kenya	To assess the knowledge, attitudes and practices of health-care providers and the community in general about the medicalization of FGM among the Abagusii	Descriptive study	In-depth interviews	Nurses	29	FGM
					Clinicians or doctors	14	
					Support staff nurses	17	
					Nurse aides	8	
					Midwives	5	
					Community health workers	2	
Ogunsiji [36] (2015)	Australia	To report the knowledge and attitude of Australian midwives towards FGM	Qualitative study	Semi-structures interviews	Midwives	11	FGM & Re-infibulation
Onuh & al. [40] (2006)	Nigeria	To determine the knowledge, attitude and practice of FGM among nurses in the ancient metropolis of Benin (urban environment) in a Nigerian state where FGM is illegal	Descriptive study	Self-administered structured questionnaire	Nurses	182	FGM
Refaat [37] (2009)	Egypt	To estimate the determinants of the practice of FGM among Egyptian physicians	Cross-sectional descriptive study	Self-administered questionnaire	Medical doctors	193	FGM
Relph & al. [43] (2013)	United Kingdom	To assess the knowledge, attitude and training on FGM amongst medical and midwifery professionals working in an area of high prevalence of FGM	Descriptive study	Questionnaire	Medical doctors	47	FGM & Re-infibulation
					Nurses-midwives	19	
					Medical/midwifery students	14	
Umar & Oche [39] (2014)	Nigeria	To identify the predictors of health-care providers practicing FGM in Sokoto, Nigeria	Cross-sectional descriptive study	Self-administered questionnaire	Female nurses	100	FGM

^a^Form of FGM: FGM (Types 1, 2, 3 and/or 4) and/or re-infibulation

### Report

This integrative review is reported following the Preferred Reporting Items for Systematic Reviews and Meta-Analyses (PRISMA) guidelines [34].

## Results

**Fig. 1:**
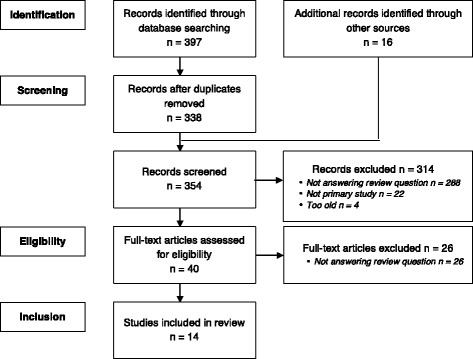
Flow diagram of search strategy

Three hundred ninety-seven (397) articles were identified through database searches, from which 59 duplicates were excluded, and 16 articles were identified through manual search of reference lists, for a total of 354 articles. Titles and abstracts were then screened to determine whether they were eligible for inclusion, and 40 full texts were examined. A total of 14 studies were included in this review. The search strategy flow diagram is presented in Fig. 1.

### Description of studies

Three (3) studies used qualitative data, nine were quantitative studies (descriptive), and two used mix-methods, using both qualitative and quantitative designs. Nine (9) studies related to the practice of FGM, two studies specifically focussed on re-infibulation, and three studies addressed both explicitly. Several studies included more than one type of health-care providers: seven studies examined the practice of nurses, seven of midwives and seven of medical doctors. Finally, a total of 10 studies were carried out in five countries where FGM is prevalent (i.e., Egypt, Sudan, Kenya, Nigeria and The Gambia), and four were undertaken in countries where FGM is not the social norm, but where women and girls from “FGM countries” immigrate (i.e., United Kingdom, Belgium, Australia and United-States of America). The summary of the articles reviewed as well as the summary of their characteristics are presented in Tables 1 and 2 respectively.

**Table 2:** Summary of the characteristics of included studies

Characteristics of included studies	Description	n	References
Type of studies	Qualitative	3	[35, 36, 48]
	Quantitative - descriptive	9	[37–44, 46]
	Mixed (qualitative and quantitative)	2	[45, 47]
Form of FGM	FGM (Types 1, 2, 3 and/or 4)	9	[37–42, 46–48]
	Re-infibulation	2	[35, 45]
	FGM & re-infibulation	3	[36, 43, 44]
Type of health-care provider	Nurses	7	[39–43, 47, 48]
	Midwives	7	[35, 36, 41, 43, 45–47]
	Physicians	7	[37, 42, 43, 47, 48] Gynaecologists: [44] Medical students: [38]
Country where the research took place	Country where FGM is prevalent	10	[35, 37–42, 46–48]
	Country of immigration (where FGM is not prevalent)	4	[36, 43–45]

### Quality assessment of studies

The quality was disparate across studies, with scores varying between 24 and 76%. In fact, the quality assessment of included studies revealed that only one study had a high methodological quality [35]. Ten (10) studies had moderate methodological limitations [36–45], two were rated as being of low quality [46, 47], and the scientific report of one study provided very limited information for the reader to assess the rigor and quality of the research and was therefore judged as being of very low quality [48].

Among the shortcomings identified in the included studies, an inconsistency was revealed about the definition used of what constitutes a health-care provider. Indeed, an author first presented the providers as being “midwives”, whereas in his methods section, he clarified that they were in reality traditional birth attendants [46], and that most of them were illiterate (63.1%). Another researcher stratified his sample according to 3 regions of the country, and equated geographical variables with cultural characteristics [37]. However, this seemingly arbitrary characterization cannot adequately represent a proxy for the culture.

### Review of evidence

The themes and sub-themes extracted from the studies about the reasons why health-care providers perform FGM or re-infibulation and the reasons why they do *not* perform FGM are presented in Table 3. These are also described in the sections below.

**Table 3:** Thematic analysis

	Themes	Sub-themes	References	
			FGM countries	Immigration countries
Reasons to perform FGM	Harm reduction vs the procedure being carried out by a traditional practitioner	To prevent unnecessary harm and reduce health complications	[35, 37, 38, 40, 41, 47, 48]	[44]
		By providing safe/hygienic conditions	[38, 47]	
		By reducing pain with anaesthesia	[38]	
	Cultural reasons	“Cultural reasons”	[39, 40, 46]	
		Convinced about the benefits of FGM	[37]	
		Trying to enhance women’s value (to do well for woman): helping the woman to maintain marriage (for husband’s sexual pleasure); beautification, completion	[35]	
		Seeing themselves as safeguards of the tradition	[48]	
	Financial reasons	For profit/for money	[35, 37, 40, 46, 47]	
		Gifts	[47]	
	Trying to satisfy the requests of the community & Community/social pressure	Responding to sociocultural requests	[35, 47]	
		Dealing with pressure from the family/community	[35, 40, 47]	
		To respond to requests as a way of demonstrating respect for cultural values and upholding customs and traditions	[47]	
	Strategy to decrease FGM practice	First step towards the prevention of the practice	[38]	
	Religious requirement		[46]	
	Legal practice	Would support a woman’s request for re-infibulation after childbirth if it was legal		[43]
Reasons *not* to perform FGM	Health complications of FGM		[35, 46]	
	Illegal practice		[46]	[36, 44, 45]
	FGM is a “bad practice”	Not a good practice	[42]	
		Anger towards the practice		[36]
	Unconvinced about the benefits of FGM		[37]	
	Not specialized for performing FGM		[37]	

#### Reasons health-care providers perform FGM, including re-infibulation

The key findings about the motivating factors for health-care providers to practice FGM and/or re-infibulation can be grouped under the following themes: for harm reduction (as compared to the procedure being carried out by a traditional practitioner), for cultural reasons, for financial reasons, and to respond to community’s request or pressure.

##### Harm reduction

A proportion of health-care providers practice FGM or re-infibulation to prevent or reduce the risks for girls and women of undergoing the procedure with a traditional practitioner [35, 37, 38, 40, 41, 44, 47, 48]. According to them, carrying out the procedure under hygienic conditions would reduce the harm for girls [38, 47]. Future health-care providers also mentioned that the medicalized procedure would reduce pain for girls, with the administration of anaesthetic medication [38]. In an exploratory study, it was even found that some midwives with a negative attitude towards FGM choose to practice re-infibulation “because someone else would do it worse” [35]. Finally, in a country like Belgium where FGM is not the social norm, 21.2% of the 333 gynaecologists surveyed agreed that FGM should be performed by a medical doctor as a harm reduction strategy [44].

##### Cultural reasons

Many health-care providers used cultural reasons to justify their practice of FGM [35, 37, 39, 40, 46, 48], such as a study in which half (52.4%) of the Egyptian physicians practicing FGM were convinced of the benefits of the tradition [37]. In a study among nurses in Nigeria, researchers found that the main predictor for practicing FGM was the ethnic group, interpreting that their practice is influenced by their cultural beliefs [39]. However, no more detail about what was considered a “cultural reason” was given by the researchers in the studies using survey instruments. In a qualitative study there was a trend among midwives to encourage women to undergo re-infibulation after childbirth, because the providers believed that it would enhance women’s value and protect their marriage, as their husband would want to divorce them if they remained de-infibulated [35]. These midwives also mentioned that re-infibulation was important for beautification and wholeness of the woman. And finally, in another study, some nurses and medical doctors explained that they saw themselves as safeguards of the FGM tradition in Kenya [48].

##### Financial reasons

Material gains have been found to be an important incentive for a proportion of health-care providers performing FGM, either midwives, nurses and physicians, mostly in the form of money [35, 37, 40, 46, 47] but also in the form of gifts [47]. Indeed, in the surveys, the financial gain was often the preferred choice selected as the reason for practicing FGM [37, 40] or was mentioned by the health-care providers [46, 47]. In one of the qualitative studies, the economic benefit of practicing re-infibulation was also a motivation given by the Sudanese midwives, but it was not mentioned spontaneously [35].

##### Responding to community’s request or pressure

A few studies identified the desire of health-care providers to satisfy the requests of the community in regards to FGM as a reason for its medicalization. In fact, answering the sociocultural requests of the Sudanese community members has been found to be pivotal for midwives to practice re-infibulation [35]. In another study, 9% of the Kenyan health-care providers interviewed mentioned responding to the “traditional/cultural demands” as a reason to perform FGM [47]. Furthermore, a proportion of health-care providers stated being under the pressure of the community to perform FGM and/or re-infibulation [35, 40, 47], for instance to demonstrate their respect for the community’s cultural values and traditions [47]. Some consider practicing FGM again in the future if they were pressured by the family to do so [40].

##### Other reasons

Only one study, in which medical students were surveyed, found that the medicalization of FGM would be a “first step towards the prevention” of the FGM practice [38]. This reason was not suggested in any other studies using close-ended questionnaires, nor did it emerge in the narratives of health-care providers in the qualitative studies. Likewise, only one study, among Sudanese midwives, reported that a religious imperative was motivating them to perform FGM [46]. Finally, one study, involving British health-care providers, found that requests for re-infibulation after childbirth would be granted if it was legal [43].

#### Reasons health-care providers do not perform FGM, including re-infibulation

Fewer studies examined the reasons why providers do not practice FGM and/or re-infibulation. The main reasons identified pertain to the risks of FGM to girls’ and women’s health, the concern about legal sanctions that might arise from performing FGM, and the conviction that FGM is a “bad practice”.

##### Health complications of FGM

Some health-care providers refuse to be involved in cutting girls because of the risks it can involve for girls and women. This was found in Sudan, where, despite the fact that the vast majority (80.9%) of the midwives “experienced FGM sometime in their life”, one-third (33.8%) of them stated they do not have the intention to practice it in the future because of possible complications [46]. In the same country, it was found that some midwives were also reluctant to perform re-infibulation, questioning the practice for the same reason [35].

##### Illegal practice

Some studies mentioned the legal liability of health-care providers as a reason for not performing FGM [36, 44–46]. This was mainly found in countries where FGM is not the norm (Belgium, Australia and United-States of America).

##### FGM as a “bad practice”

In one study, 93.2% of the Nigerian medical doctors and nurses responded that FGM was “not a good practice” [42]. However, this survey did not explore the reasons further. In another study, Australian midwives were found to have a highly negative attitude towards FGM by expressing anger towards this tradition, which could indicate that they consider FGM to be a bad practice [36].

##### Other reasons

Some providers do not perform the procedure because they do not believe that FGM is beneficial for girls. Indeed, 156 of the 193 Egyptian medical doctors that were surveyed do not practice FGM, and the reason stated by the majority of them (81.4%) is that they are “unconvinced about the benefits” of FGM [37]. The same study was also the only one that mentioned that a proportion of physicians refuse to practice FGM because they consider themselves to not have the competencies and specialization to perform this “operation” [37]. The author did not provide any more detail about this reason.

## Discussion

Despite the international human rights principles stating that every girl’s security, health and life should be protected [4, 12, 13] and the WHO statement against the medicalization of FGM [3], an increasingly alarming proportion of health-care providers continue to maintain the FGM tradition [2]. For example, in Egypt, the percentage of girls that had FGM performed by a health-care provider was 55% in 1995, and increased to 77% in 2008. An increase in the medicalization of FGM was also found in Kenya where it increased from 34–41% in one decade, i.e., between 1998 and 2008–2009. This integrative review illustrates that health-care providers have several motivations to perform FGM and re-infibulation.

The “harm reduction” rationale seems to be the main reason why some health-care providers are in favour of being involved in the medicalization of FGM. Indeed, those that subscribe to that belief feel the girls would benefit from undergoing FGM with a health-care provider, who would use aseptic techniques for the operation, as opposed to a traditional practitioner. Moreover, some argue that girls could be spared from the pain of the procedure by having access to anaesthetic and analgesic medication (where it is available), and also that health-care providers are trained to intervene in case of severe bleeding or infection. However, every provider should know that cutting and/or removing healthy body parts without medical indication is not without risks and violates medical ethics, even if done under optimal sanitary conditions. Unfortunately, it was shown that many health-care providers have poor knowledge about the health risks associated with FGM, either in countries where FGM is more frequent [37–42, 46] as well as in countries hosting immigrants [43, 45]. Therefore, this finding suggests that information and training about the risks of FGM should be given to all health-care providers caring for girls and women, including in western countries receiving immigrants.

Furthermore, the strategies aimed at eliminating the practice of FGM have largely focussed on warning about its risks for girls’ and women’s health [23]. This approach seems to have failed to reduce the prevalence of FGM, and to rather lead to an increase of its medicalization to reduce harm for girls [2, 3, 23]: more families and communities request medicalized FGM, and more health-care providers offer the service [2]. Although the population needs to be aware of the immediate and long term risks associated with FGM, this angle alone “is not sufficient to undermine a practice based on cultural beliefs and a perceived need to control women’s sexuality and fertility” [23]. Consequently, public health approaches and policies targeting FGM should be redesigned to be more comprehensive, taking into consideration the sociocultural factors related to this practice as well as the human rights principles, in addition to the health issues.

Cultural reasons were also often reported in studies, showing that many health-care providers do perform FGM for non-scientific and non-health-related reasons, such as beliefs about the preference of husbands, cultural identity and beauty criteria. Most of the studies constituting this review were assessing the motivation of providers from countries where FGM is prevalent. It is therefore not surprising that despite their professional training, they would be influenced by their own cultural group’s convictions. The fact that some of the providers either have a positive attitude towards FGM, have undergone FGM themselves or have maintained the tradition for their daughters [35, 38–41, 46, 48] indicates that it is not always obvious for them to make a distinction between their personal beliefs and their professional obligations. On the other hand, health-care providers working in countries in which FGM is not part of the culture generally seemed to have negative attitudes towards this tradition [36]. However, several researchers assumed that providers working in countries where FGM is not the norm would be against the practice. This is an important shortcoming, since some seem to show cultural relativism and therefore tolerance for practices such as FGM [43]. Future studies should then take into account the cultural beliefs of health-care providers about FGM, no matter the country where they work or come from. Health-care providers should receive appropriate training based on the content and guiding principles of the United Nations interagency statement on ending medicalization of FGM [3], in order to understand the implications of FGM for girls and women’s health and sexuality. This would ensure their professional practice adheres to the Hippocratic oath of not doing harm, which is an ethical imperative that every health-care provider should uphold.

The consideration of the financial incentive for health-care providers to perform FGM and/or re-infibulation also emerged in this review. As Toubia & Sharief reported in their review, one Egyptian doctor stated: “It [FGM] is one of those high gain low risk operations that are too lucrative to forgo unless your license is at stake” [23]. Moreover, bearing in mind that most of the FGM procedures are undertaken in low income countries, this is an important motivating factor for providers, and in particular for nurses and nurse-midwifes known to have lower salaries than medical doctors. The financial motivation should not be overlooked in high-income countries as well, and this should be explored more in-depth in future researches, particularly as it relates to cosmetic surgeries. Also, any strategy aimed at ending medicalization of FGM should take the financial aspect into consideration.

Trying to meet with the community’s expectations, and even dealing with the social pressure put upon them, are other key issues in understanding the reasons for which health-care providers perform FGM and re-infibulation. Providers need to be taught skills and given support for dealing with such requests, in order to refuse to contribute to this tradition. Likewise, professional associations should take a public stand against the practice of FGM and re-infibulation, and should disseminate their consensus statement to their members and to society at large to help reduce the community pressure on providers. For example, such statements were issued by the International Federation of Gynecology and Obstetrics [49], the Society of Obstetricians and Gynaecologists of Canada [10] and the Royal College of Obstetricians & Gynaecologists of United Kingdom [50].

Additionally, the fact that FGM is being legally banned in many countries seems to influence some health-care providers’ decisions about not to perform the intervention, whereas some others seem to allow themselves to practice FGM because no law forbids them to do so, or because the law is not enforced. It is noteworthy that the majority of governments of high prevalence countries recognise that FGM is a violation of human rights [23]. Nearly all countries where the studies included in this review took place, had legislation to prohibit the practice of FGM before the studies were undertaken: this is the case for all the Western countries, as well as most countries where FGM is commonly practiced (Egypt (2008); Sudan (2008–2009); Kenya (2001, 2011) and Nigeria (1999–2006) [2, 51]). The only exception is The Gambia, where FGM was recently outlawed (2015) [52]. Interestingly, in the other study undertaken in Sudan, as well as the studies done in Egypt, Kenya and Nigeria, the legal issue did not come up in the findings, which is another demonstration that banning the practice is insufficient in itself to end the medicalization of FGM [23]. Indeed, some health-care providers are involved in the practice despite existing laws [35, 40] and choose to take the risk of being caught, since other motivations are important for them. For example, some providers admitted to discretely performing the act within the walls of the public health-care centre where they work. And “as most of the midwives and some of the physicians seemed to be involved in and aware of the procedures taking place”, this practice seems to be hidden or even tolerated [35]. Likewise, some providers prefer to practice FGM underground, for example in their own home. Health-care providers should receive the proper information to better appropriate the law. Moreover, laws banning the practice of FGM should be reinforced by sanctioning health-care providers, either by the suspension or withdrawal of their professional licence, or by civil punitive measures (i.e., fine or imprisonment). Health institutions (hospitals and clinics) allowing or condoning the practice of FGM or re-infibulation inside their walls should also be held accountable.

Since some inconsistencies were found in categorizing some types of health-care professionals, defining what type of providers to include should be considered when studying the phenomenon of medicalization of FGM. Indeed, in some contexts such as low income countries where a shortage of adequately skilled health professionals is common [24], the distinction between a professional trained in a university and an apprentice or self-educated provider might not always be clear. Recognizing that there are different cadres of health care providers, some of whom may lack professional training or competencies, a standard definition of “medicalization” is proposed. Medicalization of FGM should refer to “health-care providers” who are professionals who have received formal training allowing them to develop adequate skills and competencies, and who are recognized by the local ministry of health as having the right to provide health care.

The studies included in this review help elucidate the medicalization phenomenon – 9 of them were undertaken in countries with not only a high proportion of girls and women having undergone FGM, but also with a high prevalence of medicalization of FGM, including Egypt (77%), Sudan (55%), Kenya (42%), and Nigeria (28%) [2]. The Gambia is an exception since despite the high prevalence of FGM [1], medicalization is not widely practiced in this country [2]. However, the study done there showed that 42.5% of the 468 nurses surveyed embraced the continuation of FGM, and 42.9% of them think that medicalization of FGM is safer than when it is performed by a traditional practitioner [41]. These findings are of great concern and show that an increasing number of health-care providers could eventually perform FGM in this context. Moreover, no studies were found from countries where the medicalization phenomenon is present, such as in Guinea, where the prevalence of FGM is as high as 97% [1], and where 27% of FGM is reported to be done by health-care providers [2]. Since communities maintain or adopt the practice of FGM mainly for sociocultural reasons [2], more research is needed in different regions where health-care providers perform FGM, in order to tailor strategies to end medicalization of FGM to each context. As 4 studies were undertaken in countries hosting immigrants from practicing nations (United Kingdom, Belgium, Australia, United-States of America) and revealed that a number of health-care providers do perform some form of FGM in these parts of the world also, it is clear that the phenomenon of medicalization of FGM is a global problem. Therefore, it should be recognized that medicalization can be practiced by health-care providers throughout the world.

### Limitations of the review

Our findings have several limitations. First, the results of this review were limited by the fact that most of the available studies were descriptive, in the form of quantitative surveys with pre-determined answer choices. Therefore, this suggests the pressing need to develop robust, in-depth qualitative studies, as well as quantitative studies that specifically focus on this topic rather than embedding questions on medicalization in surveys related to other topics.

Also, this review identified a relatively small number of studies (n = 14), with methodological limitations in nearly all the studies. Furthermore, although the “STROBE Statement” is a useful tool to improve the reporting of observational studies, it was not designed to assess quality. Therefore, a checklist for quality assessment of survey studies is needed.

Because of the paucity of studies that could be included in this review, the findings were not analysed by the type of health-care providers, nor by the sex of the providers. Additionally, the lack of information in many studies made it difficult, and even virtually impossible, to specifically distinguish the motivations of the providers according to the different types of FGM (i.e., types 1–4 and re-infibulation) they perform. This should be taken into account in future studies, since this exploration could reveal different viewpoints about the medicalization of FGM. The rising trend of “symbolic circumcision” should also be accounted for, since it is increasingly considered as an “alternative to more severe forms of cutting” [2] (but it is nonetheless a form of mutilation as per WHO). The findings of this review were not distinguished according to the types of settings in which health-care providers perform FGM or re-infibulation. Different contexts might show different motivating factors for the practice. Also, because the tradition of FGM has different meanings among diverse sociocultural groups, future studies should consider these nuances [4].

Searches for studies were carried out in the main pertinent databases as well as in the grey literature. However, unpublished research findings were not looked for, which would have allowed to complete this systematic review of the literature. Finally, since the main literature can be found in English, a keyword search in other languages was not undertaken. However, a search in languages such as in Arabic (which is a major language spoken in East Africa) and French (which is a major language spoken in West Africa and in some Western countries) would have potentially generated some additional articles. Nevertheless, no study was rejected because of the language.

## Conclusion

Many international organisations, such as the World Health Organization (WHO), the United Nations Children’s Fund (UNICEF), the United Nations Population Fund (UNFPA) and the United Nations Development Programme (UNDP), are jointly working for the eradication of the female genital mutilation tradition [5]. This study is the first review that explores the reasons related to the involvement of health-care providers in the medicalization of female genital mutilation, either in FGM-prevalent settings and countries hosting immigrants. The available findings mainly suggest that health-care providers need more information and training in order to revert these harmful practices.

Since not many studies have explored the reasons for which health-care providers practice medicalization of FGM, and since several studies had methodological limitations, more research is needed to tackle this complex phenomenon and to guide efforts to eradicate FGM around the world. This would ensure a deeper understanding of the phenomenon and richer information for different contexts in order to adequately tailor strategies, programmes, guidelines and trainings for health-care providers to end the medicalization of FGM.

## Additional files

Additional file 1: Search Strategy. (DOCX 78 kb)

Additional file 2: Critical Appraisal Skills Programme (CASP) Qualitative Research Checklist – modified. (DOCX 133 kb)

Additional file 3: STROBE (Strengthening the Reporting of Observational studies in Epidemiology) Statement – modified: Survey Design Quality Assessment Checklist. (DOC 96 kb)

## Abbreviations

CASP: Critical appraisal skills programme
CINAHL: Cumulative index to nursing and allied health literature
FGM: Female genital mutilation
PRISMA: Preferred reporting items for systematic reviews and meta-analyses
STROBE: Strengthening the reporting of observational studies in epidemiology
UNDP: United Nations Development Programme
UNFPA: United Nations Population Fund
UNICEF: United Nations Children’s Fund
WHO: World Health Organization
WHOLIS: World Health Organization Library & Information Networks for Knowledge Database
