# Supplementary material for: Understanding the motivations of health-care providers in performing female genital mutilation: an integrative review of the literature
Source: Reprod Health. 2017 Mar 23;14:46. doi: 10.1186/s12978-017-0306-5 (PMC5364567; doi:10.1186/s12978-017-0306-5)
Supplement: Supplementary file 1 — Search Strategy. (DOCX 78 kb) [file 12978_2017_306_MOESM1_ESM.docx]

**PubMed Search Strategy**

| **#** | **Searches** | **Results** |
| --- | --- | --- |
| 1 | (female genital mutilation[Title/Abstract] OR female genital cutting[Title/Abstract] OR infibulation[Title/Abstract] OR clitoridectomy[Title/Abstract] OR reinfibulation[Title/Abstract]) | 1104 |
| 2 | Circumcision, Female[MeSH Terms] | 1016 |
| 3 | #1 OR #2 | 1499 |
| 4 | (Healthcare professionals[Title/Abstract] OR Health providers[Title/Abstract] OR Midwives[Title/Abstract] OR Nurses[Title/Abstract] OR Physicians[Title/Abstract] OR doctors[Title/Abstract]) | 399456 |
| 5 | Health Personnel[MeSH Terms] | 422803 |
| 6 | (Medicalization[Title/Abstract] OR Medicalization[Title/Abstract]) | 1003 |
| 7 | #4 OR #5 OR #6 | 718084 |
| 8 | #3 AND #7 | 243 |
| 9 | Filters: Years 2001-2016 | **162** |

**CINAHL EBSCO host Search Strategy**

| **#** | **Searches** | **Results** |
| --- | --- | --- |
| 1 | (MH "Circumcision, Female") OR "female genital mutilation OR female genital cutting OR infibulation OR clitoridectomy OR reinfibulation" | 975 |
| 2 | TI Healthcare professionals OR Health providers OR Midwives OR Nurses OR Physicians OR Doctors | 548489 |
| 3 | AB Healthcare professionals OR Health providers OR Midwives OR Nurses OR Physicians OR Doctors | 557287 |
| 4 | TI Medicalization OR Medicalization | 595 |
| 5 | AB Medicalization OR Medicalization | 595 |
| 6 | #2 OR #3 OR #4 OR #5 | 558373 |
| 7 | #1 AND #6 | 253 |
| 8 | Filters: Years 2001-2016 | **235** |
