# Supplementary material for: Understanding the motivations of health-care providers in performing female genital mutilation: an integrative review of the literature
Source: Reprod Health. 2017 Mar 23;14:46. doi: 10.1186/s12978-017-0306-5 (PMC5364567; doi:10.1186/s12978-017-0306-5)
Supplement: Supplementary file 2 — Critical Appraisal Skills Programme (CASP) Qualitative Research Checklist – modified. (DOCX 133 kb) [file 12978_2017_306_MOESM2_ESM.docx]

**Critical Appraisal Skills Programme (CASP) Qualitative Research Checklist – modified**

| **Screening questions** | **Did the researcher(s)…** | **Score*** | **Comments** |
| --- | --- | --- | --- |
| **1. Was there a clear statement of the aims of the research?** | *state what the goal of the research was ?* |  |  |
|  | *state why it is important ?* |  |  |
|  | *explained its relevance ? [research gap]* |  |  |
| **2. Is a qualitative methodology appropriate?** | *seek to interpret or illuminate the actions and/or subjective experiences of research participants* |  |  |
| **Is it worth continuing?** |  |  |  |
| **Detailed questions** | **Did the researcher(s)…** | **Score*** | **Comments** |
| *Appropriate research design*  **3. Was the research design appropriate to address the aims of the research?** | *justify the research design (e.g. have they discussed how they decided which methods to use?)* |  |  |
| *Sampling*  **4. Was the recruitment strategy appropriate to the aims of the research?** | *explain how the participants were selected?* |  |  |
|  | *explain why the participants they selected were the most appropriate to provide access to the type of knowledge sought by the study?* |  |  |
|  | *discuss about recruitment (e.g. why some people chose not to take part)* |  |  |
| Data collection  **5. Were the data collected in a way that addressed the research issue?** | *was the setting for data collection justified?* |  |  |
|  | *is it clear how data were collected (e.g. focus group, semi-structured interview, etc.) ?* |  |  |
|  | *justify the methods chosen?* |  |  |
|  | *make the methods explicit (e.g. for interview method, is there an indication of how interviews were conducted, did they used a topic guide?)* |  |  |
|  | *if methods were modified during the study : has the researcher explained how and why ?* |  |  |
|  | *is the form of data is clear (e.g. tape recordings, video material, notes, etc.)?* |  |  |
|  | *discuss saturation of data?* |  |  |
| *Reflexivity (research partnership relations/ recognition of researcher bias)*  **6. Has the relationship between researcher and participants been adequately considered?** | *critically examined their own role, potential bias and influence during :*  *- formulation of research questions*  *- date collection, including sample recruitment and choice of location* |  |  |
|  | *how did the researcher respond to events during the study and whether they considered the implications of any changes in the research design?* |  |  |

| **Detailed questions** | **Did the researcher(s)…** | **Score*** | **Comments** |
| --- | --- | --- | --- |
| *Ethical Issues*  **7. Have ethical issues been taken into consideration?** | *is there are sufficient details of how the research was explained to participants for the reader to assess whether ethical standards were maintained* |  |  |
|  | *discuss issues raised by the study (e.g. issues around informed consent or confidentiality or how they have handled the effects of the study on the participants during and after the study)* |  |  |
|  | *has approval been sought from the ethics Committee* |  |  |
| *Data Analysis*  **8. Was the data analysis sufficiently rigorous?** | *is there an in-depth description of the analysis process* |  |  |
|  | *if thematic analysis is used: is it clear how the categories/ themes were derived from the data?* |  |  |
|  | *explain how the data presented were selected from the original sample to demonstrate the analysis process?* |  |  |
|  | *present sufficient data to support the findings?* |  |  |
|  | *to what extent contradictory data are taken into account?* |  |  |
|  | *critically examine their own role, potential bias and influence during analysis and selection of data for presentation?* |  |  |
| *Findings*  **9. Is there a clear statement of findings?** | *are the findings explicit?* |  |  |
|  | *is there an adequate discussion of the evidence both for and against the researcher’s arguments*? |  |  |
|  | *discuss the credibility of their findings (e.g. triangulation, respondent validation, more than one analyst)?* |  |  |
|  | *are the findings discussed in relation to the original research questions?* |  |  |
| *Value of the research*  **10. Does the research add value to the review?** | *discuss the contribution the study makes to existing knowledge or understanding (e.g. do they consider the findings in relation to current practice or policy or relevant research-based literature)?* |  |  |
|  | *identify new areas where research is necessary?* |  |  |
|  | *discuss whether or how the findings can be transferred to other populations or considered other ways the research may be used?* |  |  |
|  | *mention the possible bias/limits of the study?* |  |  |
| **TOTAL SCORE** |  | **___ / ___** | **____ %** |

* Legend : 1 = entirely fills the criteria ; ½ = partially fills the criteria ; 0 = does not fill the criteria; NA : non applicable

Source: Gray M et al., Better Value Healthcare Institute (Oxford, UK). <http://www.casp-uk.net/#!casp-tools-checklists/c18f8>
