# Supplementary material for: Understanding the motivations of health-care providers in performing female genital mutilation: an integrative review of the literature
Source: Reprod Health. 2017 Mar 23;14:46. doi: 10.1186/s12978-017-0306-5 (PMC5364567; doi:10.1186/s12978-017-0306-5)
Supplement: Supplementary file 3 — STROBE (Strengthening the Reporting of Observational studies in Epidemiology) Statement – modified: Survey Design Quality Assessment Checklist. (DOC 96 kb) [file 12978_2017_306_MOESM3_ESM.doc]

**STROBE (Strengthening the Reporting of Observational studies in Epidemiology) Statement – modified**

**Survey Design Quality Assessment Checklist**

|  | Item No | Did the researcher(s)… | Score | Comments |
| --- | --- | --- | --- | --- |
| **Title and abstract** | 1a | indicate the study’s design with a commonly used term in the title or the abstract? |  |  |
|  | 1b | provide in the abstract an informative and balanced summary of what was done and what was found? |  |  |
| Introduction | | |  |  |
| Background/rationale | 2 | explain the scientific background and rationale for the investigation being reported? |  |  |
| Objectives | 3 | state specific objectives? |  |  |
| Methods | | |  |  |
| Study design | 4 | present key elements of study design early in the paper? |  |  |
| Setting | 5 | describe the setting, locations, and relevant dates, including periods of recruitment and data collection? |  |  |
| Participants | 6 | give the eligibility criteria, and the sources and methods of selection of participants? |  |  |
| Data sources | 7 | for each variable of interest, give sources of data and details of methods of assessment? Describe comparability of assessment methods if there is more than one group? |  |  |
| Bias | 8 | describe any efforts to address potential sources of bias? |  |  |
| Study size | 9 | explain how the study size was arrived at? |  |  |
| Quantitative variables | 10 | explain how quantitative variables were handled in the analyses. If applicable, describe which groupings were chosen and why |  |  |
| Statistical methods | 11a | Describe all statistical methods? |  |  |
|  | 11b | Explain how missing data were addressed? |  |  |
|  | 11c | If applicable, describe analytical methods taking account of sampling strategy? |  |  |
| Ethical considerations [added criterion] | 12 | Report that the approval was sought from ethics Committee? |  |  |

|  | Item No | Did the researcher(s)… | Score | Comments |
| --- | --- | --- | --- | --- |
| **Results** |  |  |  |  |
| Participants | 13a | Report numbers of individuals at each stage of study—eg numbers potentially eligible, examined for eligibility, confirmed eligible, included in the study, completing follow-up, and analysed? |  |  |
|  | 13b | Give reasons for non-participation at each stage? |  |  |
| Descriptive data | 14a | Give characteristics of study participants (eg demographic, clinical, social)? |  |  |
|  | 14b | Indicate number of participants with missing data for each variable of interest? |  |  |
| Main results | 15 | Give unadjusted estimates and, if applicable, confounder-adjusted estimates and their precision (eg, 95% confidence interval)? Make clear which confounders were adjusted for and why they were included? |  |  |
| Other analyses | 16 | Report other analyses done—eg analyses of subgroups and interactions, and sensitivity analyses? |  |  |
| Discussion | | |  |  |
| Key results | 17 | Summarise key results with reference to study objectives? |  |  |
| Limitations | 18 | Discuss limitations of the study, taking into account sources of potential bias or imprecision? Discuss both direction and magnitude of any potential bias? |  |  |
| Interpretation | 19 | Give a cautious overall interpretation of results considering objectives, limitations, multiplicity of analyses, results from similar studies, and other relevant evidence? |  |  |
| Generalisability | 20 | Discuss the generalisability (external validity) of the study results? |  |  |
| Other information | | |  |  |
| Funding | 21 | Give the source of funding and the role of the funders for the present study and, if applicable, for the original study on which the present article is based? |  |  |
| **TOTAL SCORE** |  |  | **____ / ____** | **____ %** |

* Legend : 1 = entirely fills the criteria ; ½ = partially fills the criteria ; 0 = does not fill the criteria; NA : non applicable

Source: Vandenbroucke, J.P. et al. (2007). "Strengthening the Reporting of Observational Studies in Epidemiology (STROBE): Explanation and Elaboration."

PLOS Med **4**(10): e297.
